# High Fall Risk Associated With Memory Deficit and Brain Lobes Atrophy Among Elderly With Amnestic Mild Cognitive Impairment and Mild Alzheimer’s Disease

**DOI:** 10.3389/fnins.2022.896437

**Published:** 2022-06-08

**Authors:** Shuyun Huang, Xinhan Zhou, Yajing Liu, Jiali Luo, Zeping Lv, Pan Shang, Weiping Zhang, Biqing Lin, Qiulan Huang, YanYun Feng, Wei Wang, Shuai Tao, Yukai Wang, Chengguo Zhang, Lushi Chen, Lin Shi, Yishan Luo, Vincent C. T. Mok, Suyue Pan, Haiqun Xie

**Affiliations:** ^1^Department of Neurology, First People’s Hospital of Foshan, Foshan, China; ^2^Department of Neurology, Nanfang Hospital, Southern Medical University, Guangzhou, China; ^3^Department of Imaging, First People’s Hospital of Foshan, Foshan, China; ^4^National Research Center for Rehabilitation Technical Aids, Rehabilitation Hospital, Beijing, China; ^5^Dalian Key Laboratory of Smart Medical and Health, Dalian University, Dalian, China; ^6^Department of Imaging and Interventional Radiology, The Chinese University of Hong Kong, Shatin, Hong Kong SAR, China; ^7^BrainNow Research Institute, Shenzhen, China; ^8^Division of Neurology, Department of Medicine and Therapeutics, The Chinese University of Hong Kong, Shatin, Hong Kong SAR, China

**Keywords:** fall risk, memory deficit, medial temporal lobe atrophy, amnestic mild cognitive impairment, mild Alzheimer’s disease

## Abstract

**Objectives:**

This study aimed to primarily examine the association between memory deficit and increased fall risk, second, explore the underlying neuroanatomical linkage of this association in the elderly with aMCI and mild AD.

**Methods:**

In this cross-sectional study, a total of 103 older adults were included (55 cognitively normal, CN; 48 cognitive impairment, CI, elderly with aMCI, and mild AD). Memory was assessed by the Auditory Verbal Learning Test (AVLT). Fall risk was evaluated by the Timed Up and Go (TUG) Test, heel strike angles, and stride speed, which were collected by an inertial-sensor-based wearable instrument (the JiBuEn™ gait analysis system). Brain volumes were full-automatic segmented and quantified using AccuBrain^®^ v1.2 from three-dimensional T1-weighted (3D T1W) MR images. Multivariable regression analysis was used to examine the extent of the association between memory deficit and fall risk, the association of brain volumes with memory, and fall risk. Age, sex, education, BMI, and HAMD scores were adjusted. Sensitivity analysis was conducted.

**Results:**

Compared to CN, participants with aMCI and mild AD had poorer cognitive performance (*p* < 0.001), longer TUG time (*p* = 0.018), and smaller hippocampus and medial temporal volumes (*p* = 0.037 and 0.029). In the CI group, compared to good short delayed memory (SDM) performance (AVLT > 5), the elderly with bad SDM performance (AVLT ≤ 3) had longer TUG time, smaller heel strike angles, and slower stride speed. Multivariable regression analysis showed that elderly with poor memory had higher fall risk than relative good memory performance among cognitive impairment elderly. The TUG time increased by 2.1 s, 95% CI, 0.54∼3.67; left heel strike angle reduced by 3.22°, 95% CI, −6.05 to −0.39; and stride speed reduced by 0.09 m/s, 95% CI, −0.19 to −0.00 for the poor memory elderly among the CI group, but not found the association in CN group. In addition, serious medial temporal atrophy (MTA), small volumes of the frontal lobe and occipital lobe were associated with long TUG time and small heel strike angles; small volumes of the temporal lobe, frontal lobe, and parietal lobe were associated with slow stride speed.

**Conclusion:**

Our findings suggested that memory deficit was associated with increased fall risk in the elderly with aMCI and mild AD. The association might be mediated by the atrophy of medial temporal, frontal, and parietal lobes. Additionally, increased fall risk, tested by TUG time, heel stride angles, and stride speed, might be objective and convenient kinematics markers for dynamic monitoring of both memory function and fall risk.

## Introduction

Cognitive impairment and falls are leading causes of low health-related quality of life in the elderly. Cognitive impairment was an independent risk factor for falls (Baydan et al., 2019). There has been a growing interest in motoric cognitive risk syndrome (MCR), characterized as a cognitive disorder and motor dysfunction in older people (Verghese et al., 2013, 2014).

Gait disorder was one of the determinants of motor dysfunction. It was prevalent in the development stages of dementia (Beauchet et al., 2008; Verghese et al., 2008; Montero-Odasso et al., 2012). Allali et al. (2015) reported that gait disorder was parallel with cognitive decline from mild cognitive impairment (MCI)to moderate dementia. A cross-sectional study showed that stride speed was slow among individuals with early cognitive impairment (Knapstad et al., 2019). Eggermont et al. (2010) suggested that gait performance measured by the Timed Up and Go (TUG) test, a screening tool to assess fall risk (Society et al., 2001; Persons and Society, 2011), was worse in the AD group compared to normal controls.

Currently, fall risk assessment tools used for the elderly did not show sufficiently high validity. A review revealed that the TUG test is a standard screening tool to identify the elderly at risk of falling. While it has limited ability to predict falls in community-dwelling elderly and should not be used in isolation (Barry et al., 2014). Thereupon, stride speed has been highlighted as a potentially suitable screening tool for identifying individuals with high fall risk (Kyrdalen et al., 2019). Several studies have demonstrated an association between slow stride speed and increased fall risk in normal older adults (Abellan et al., 2009; Middleton et al., 2015; Hsu et al., 2017). Among the elderly with mild cognitive impairment (MCI), slow stride speed was also associated with increased fall risk (Adam et al., 2021).

Moreover, most falls occurred during walking. The impaired control of balance during walking is contributed to falls. Heel stride angle, reflected by the lifting of the foot, might be an indicator of balance. Ginis et al. (2017) found that the heel stride angle was robustly reduced in Parkinson’s disease patients than in controls. However, the literature on heel stride angle in elderlies with Alzheimer’s disease (AD) is relatively sparse. Hence, more parameters used together would better evaluate fall risk rather than a single parameter.

The common factors leading to falls include dyskinesia, cognitive impairment, medications, etc. Recently, given memory deficit was the main clinical hallmark of aMCI and AD, memory deficit and falls in the elderly have gained more interest. A cross-sectional study evaluated fall risk by TUG test among older community-dwellers without dementia. Results suggested that increased TUG time was associated with declined memory (Beauchet et al., 2014). Inconsistent with this finding, one small sample study revealed that poor visuospatial function rather than memory deficit was associated with increased fall risk among the elderly with MCI and AD (Ansai et al., 2017). In these studies, memory deficits were evaluated by brief screening tests, which were less accurate to reflect the memory function compared to the comprehensive neuropsychology battery tests. It is preferable to apply neuropsychology tests of cognitive domains and explore the relationship between fall risk and these more reliable mental measurements.

In addition, the neural mechanism of fall risk relevant to cognitive impairment was still unclear. The temporal lobe and hippocampal atrophy have been demonstrated to be associated with memory deficit. To date, few studies have explored if these brain volumes are related to falling risk in aMCI and mild AD elderly.

Taken together, we hypothesized that high fall risk is associated with memory deficit in aMCI and mild AD in older adults. Thereby, we aimed to primarily examine the association between memory deficit and increased fall risk, second, explore the underlying neuroanatomical linkage of this association in the elderly with aMCI and mild AD in this study.

## Materials and Methods

### Participants

In this study, 48 participants with cognitive impairment (CI) were recruited who were diagnosed with aMCI or mild AD at neurological clinics of First People’s Hospital of Foshan between October 2018 and December 2019. Fifty-five cognitive normal (CN) older adults with matching demographic information (age, sex, and education level) were enrolled in the communities. Demographic characteristics, medical history, and Hamilton Depression Scale (HAMD) scores were collected in face-to-face interviews. Clinicians performed a physical examination for all participants, including a neurological exam. Ethics approval was obtained from the Research Ethics Board of the First People’s Hospital of Foshan and written informed consent was obtained from the participants at enrollment.

Inclusion criteria of the aMCI (Petersen, 2004) were as follows: (1) subjective cognitive complaint, preferably confirmed by an informant; (2) Memory domain impairment with or without other cognitive domains of decline. Abnormal objective cognitive function impairment (including global cognitive function and cognitive domains) was identified by a cut-off of 1.5 SD below education and age matched-specific norms; (3) preserved activities of daily living were confirmed by a clinician’s interviews; (4) Global Clinical Dementia Rating (CDR) = 0.5 (Morris, 1993).

The mild AD diagnostic criteria were as follows: (1) diagnosed according to Diagnostic and Statistical Manual of Mental Disorders, Fourth Edition, revised (DSM-IV-R); (2) CDR = 1. The normal cognitive participants were recruited according to the criteria:(1) cognitively normal, verified by an informant; (2) CDR = 0.

Exclusion criteria for all the participants are as follows: (1) illiteracy; (2) any neurologic disorder and other systematic diseases that would likely contribute to cognitive and motor deficits (history of stroke, Parkinson’s disease, epilepsy, brain trauma, etc.), active rheumatic and orthopedic diseases that affect lower limbs, history of knee/hip replacement; (3) use of neuroleptics or benzodiazepines, and psychiatric comorbidity (e.g., significant depressive/anxiety). MRI exclusion criteria included standard contraindications: (1) claustrophobia and (2) surgically implanted metal devices.

**FIGURE 1:**
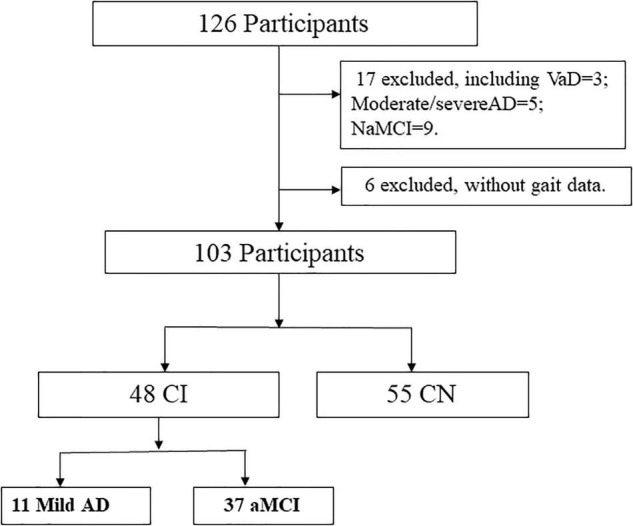
Flow diagram of participants.

From 126 participants initially recruited, we excluded 23 cases because of the diagnosis as vascular dementia (VaD), moderate/severe AD, non-amnestic MCI, or without gait data at the time of sorting data (Figure 1).

### Neuropsychological Assessment

Global cognition was assessed using the standardized Mini-mental State Examination (MMSE) (Folstein et al., 1975). A neuropsychological test battery was carried out. Memory was assessed by Auditory Verbal Learning Test (AVLT) (Guo, 2001), which included immediate memory (IM, AVLT-1) and short delay memory (SDM, 5-min recalls, AVLT-4). The language was assessed with Boston Naming Test (BNT), executive function with Stroop Color-Word Test (SCWT), attention with Symbol Digit Modalities Test (SDMT), and visual-Spatial with Clock Drawing Test (CDT). The score of neuropsychological tests was converted to a standard score in the application. MCI was identified by a cut-off of 1.5 SD. Clinical Dementia Rating Scale (CDR) was administered as well.

### Gait Measurements

**FIGURE 2:**
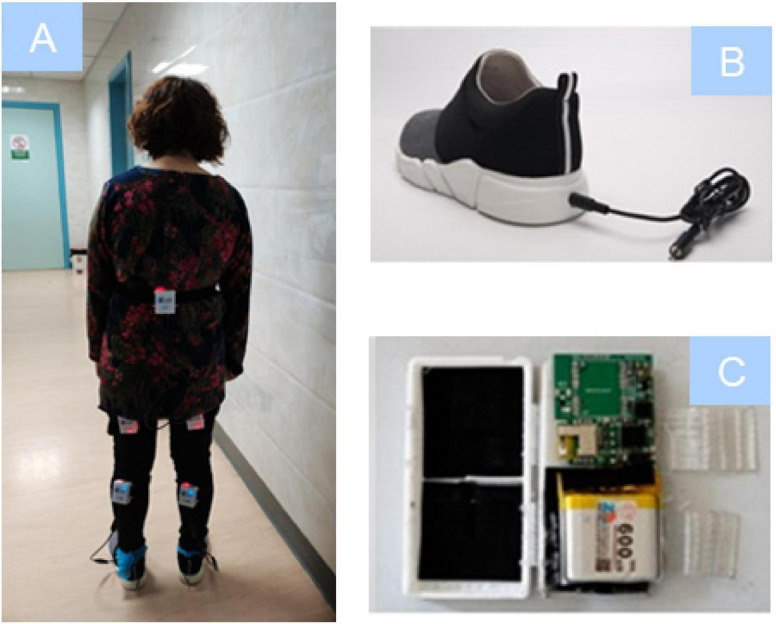
The modules **(A,C)** and shoes **(B)** of JiBuEn™ wearable devices.

Kinetic parameters were collected by the JiBuEn™ gait analysis system. The method comprises wearable devices of shoes and modules with the inertial Micro-Electro-Mechanical Systems sensors fixed under the shoe bottom, behind loin, the upper and lower limbs, collecting motion signals and transmitting them to a computer (Figure 2). The high-order, low-pass filter, and hexahedral calibration techniques are employed in data preprocessing, which reduces high-frequency noise interference and installation errors produced by sensor devices (Tao et al., 2018; Xie et al., 2019; Gao et al., 2021).

Free Walking test and TUG test were implemented. Participants needed to walk 30 s at their usual speed on a walkway in a quiet, well-lit room wearing specially made shoes for the Free Walking test. Start and endpoints were marked on the floor 1 m from the walkway end to avoid recording acceleration and deceleration phases. The parameters of stride speed, cadence, and heel strike angles came from the Free Walking test. TUG test measures in seconds, which is the time needed to rise from a chair, walk three meters, turn around, and return to a seated position at a faster speed. Three times were tested. In this study, TUG time was the average of the three times. Longer TUG time, small heel strike angles, and slow stride speed reflect higher fall risk.

### MR Imaging Technique

All participants underwent MRI scanning using an imaging system (GE Discovery MR750w 3.0 T). Brain MRI scans were acquired 1 month after completing the clinical examination and gait assessment. Three-dimensional sagittal T1weighted (3DT1W) images were acquired with the following parameters: repetition time (TR) = 8.14 ms, echo time (TE) = 3.17 ms, flip angle (FA) = 12°, inversion time (TI) = 450 ms, matrix = 256 × 256, FOV = 256 × 256 mm^2^, number of slices = 188, slice thickness = 1.0 mm, slice gap = 0 mm, spatial resolution = 1 × 1 × 1 mm^3^, and acquisition time = 3 min 46 s. Additional scans were collected in all participants, including T2-FLAIR, Arterial Spin Labeling, and Susceptibility Weighted Imaging.

The 3DT1W MRI images were automatically analyzed using AccuBrain^®^ v1.2 to quantify volumetries (Abrigo et al., 2018). The system used Multi-atlas non-rigid registration scheme. AccuBrain^®^ v1.2 system automatically segmented brain regions and obtained absolute volume (AV), relative volume (RV), and percentile of different brain regions. AV is the actual volume of the brain region (in ml). RV is the ratio of the AV to the individual’s intracranial volume (ICV). Brain lobe atrophy ratio (AR) is the ratio of cerebrospinal fluid (CSF) volume to brain parenchyma in a particular lobe. In this study, the volumes of the hippocampus, temporal lobe, etc., are RV. Quantitative medial temporal atrophy (QMTA) was calculated as the volume ratio between the inferior lateral ventricle and hippocampus.

### Statistical Analysis

The comparison of demographic characteristics, scores of neuropsychological assessments, gait parameters, and brain volumes were appropriate between the two groups using *t*-test or chi-square test. Multivariable regression analyses were used to estimate the effect values (β) and 95% confidence intervals (CI) to examine the extent of the association between cognitive tests and gait parameters, the association between brain volumes and memory deficit, and the association between brain volumes and gait parameters. Age, sex, education, BMI, and HAMD scores were adjusted. Sensitivity analysis was administered. Analyses were conducted by the statistical software packages R^1^ (The R Foundation) and Empower Stats (^2^ X&Y solutions, Inc., Boston, MA, United States). All the *p* < 0.05 were considered statistically significant.

## Results

### Participants Characteristics

The flowchart in Figure 1 illustrates the process of sample selection.

The descriptive characteristics of the study population are shown in Table 1 and Supplementary Table 1. Compared to CN, participants with aMCI and mild AD had poorer cognitive performance (*p* < 0.001), longer TUG time (*p* = 0.018), and smaller hippocampus and medial temporal volumes (*p* = 0.037 and 0.029). In the CI group, poor memory participants (low tertile of AVLT-4) had longer TUG time (12.30 vs. 10.31 s), smaller heel strike angles (30.37 vs. 33.51°), and slower stride speed (0.86 vs. 0.95 m/s) than good memory participants (high tertile of AVLT-4). Variables of sex, age, education, BMI, HAMD, hypertensive history, and diabetes history did not differ between the two groups (*p* > 0.05).

**TABLE 1:** Characteristics of participants.

	Total (*N* = 103)	Group CI (*N* = 48)	Group CN (*N* = 55)	*p* ^§^
**Demographic Characteristics**				
Age, years	66.4 ± 4.7	65.7 ± 5.2	67.1 ± 4.1	0.127
Male, n (%)	36 (34.9)	21 (43.8)	15 (27.3)	0.080
Education, n (%)				0.553
Primary school	28 (27.2)	11 (22.9)	17 (30.9)	
Secondary school	33 (32.0)	15 (31.3)	18 (32.7)	
Above secondary school	42 (40.8)	22 (45.8)	20 (36.4)	
BMI, kg/m^2^	23.2 ± 2.8	23.2 ± 3.0	23.3 ± 2.6	0.820
HAMD, score	6.87 ± 3.58	7.42 ± 3.75	6.40 ± 3.39	0.151
Hypertensive history, n (%)	37 (35.9)	17 (35.4)	20 (36.4)	0.920
Diabetes history, n (%)	13 (12.6)	6 (12.5)	7 (12.7)	0.972
**Neuropsychological Assessment**				
MMSE, score	**25.8 ± 3.0**	**24.4 ± 3.5**	**27.0 ± 1.8**	**<0.001**
AVLT-1 (continuous)	**4.0 ± 1.6**	**3.3 ± 1.2**	**4.7 ± 1.6**	**<0.001**
AVLT-1 tertiles, n (%)				**0.005**
Low	**31 (30.1)**	**21 (43.8)**	**10 (18.2)**	
High	**72 (69.9)**	**27 (56.2)**	**45 (81.8)**	
AVLT-4 (continuous)	**5.2 ± 2.4**	**3.7 ± 2.2**	**6.4 ± 1.9**	**<0.001**
**AVLT-4 tertiles, n (%)**				**<0.001**
Low (≤3)	**22 (21.4)**	**20 (41.7)**	**2 (3.6)**	
Middle (3< and ≤5)	**29 (28.2)**	**16 (33.3)**	**13 (23.6)**	
High (>5)	**52 (50.5)**	**12 (25.0)**	**40 (72.7)**	
BNT, score	**20.5 ± 4.6**	**17.8 ± 4.8**	**23.0 ± 2.8**	**<0.001**
SCWT, score	**18.1 ± 14.8**	**23.1 ± 19.2**	**13.8 ± 7.0**	**<0.001**
SDMT, score	**32.6 ± 10.8**	**27.1 ± 10.6**	**37.3 ± 8.6**	**<0.001**
CDT, score	**8.0 ± 2.5**	**6.6 ± 2.7**	**9.2 ± 1.4**	**<0.001**
**Gait Parameters**				
^※^TUG time, s	**10.90 ± 2.23**	**11.44 ± 2.38**	**10.41 ± 1.99**	**0.018**
^※^Heel strike angles, degrees				
Left	30.93 ± 3.70	30.23 ± 3.83	31.55 ± 3.50	0.069
Right	31.11 ± 3.95	30.85 ± 4.12	31.33 ± 3.82	0.542
^※^Stride speed, m/s				
Left	0.90 ± 0.13	0.89 ± 0.13	0.91 ± 0.12	0.099
Right	0.90 ± 0.13	0.89 ± 0.13	0.91 ± 0.12	0.099
^※^Cadence, steps/min				
Left	98.82 ± 8.12	98.52 ± 8.38	99.07 ± 7.95	0.820
Right	98.82 ± 8.12	98.52 ± 8.38	99.07 ± 7.95	0.820
**Brain Volumes**				
White matter hyperintensity	0.21 ± 0.37	0.25 ± 0.52	0.17 ± 0.17	0.272
Cerebellum	9.29 ± 0.60	9.25 ± 0.56	9.32 ± 0.63	0.570
Hippocampus	0.49 ± 0.04	0.48 ± 0.05	0.50 ± 0.04	0.072
Left Hippocampus	**0.24 ± 0.02**	**0.24 ± 0.03**	**0.25 ± 0.02**	**0.037**
Right Hippocampus	0.25 ± 0.02	0.25 ± 0.02	0.25 ± 0.02	0.168
MTA	**34.38 ± 9.51**	**36.14 ± 10.14**	**32.81 ± 8.71**	**0.029**
Left MTA	**35.42 ± 9.87**	**37.40 ± 10.90**	**33.66 ± 8.58**	**0.036**
Right MTA	33.40 ± 10.35	34.98 ± 10.61	32.00 ± 10.00	0.053
Left Frontal lobe	6.06 ± 0.36	6.07 ± 0.34	6.05 ± 0.38	0.784
Right Frontal lobe	6.06 ± 0.36	5.93 ± 0.32	5.95 ± 0.39	0.767
Left Temporal lobe	3.82 ± 0.26	3.81 ± 0.29	3.83 ± 0.24	0.170
Right Temporal lobe	3.78 ± 0.28	3.74 ± 0.33	3.82 ± 0.23	0.687
Left Occipital lobe	2.28 ± 0.24	2.25 ± 0.25	2.31 ± 0.22	0.273
Right Occipital lobe	1.95 ± 0.28	1.97 ± 0.31	1.92 ± 0.24	0.431
Left Parietal lobe	2.84 ± 0.24	2.83 ± 0.26	2.85 ± 0.24	0.750
Right Parietal lobe	2.79 ± 0.29	2.77 ± 0.31	2.80 ± 0.27	0.623

*BMI, Body Mass Index. Values of P < 0.05 are bold. Bold fonts indicate they had statistical significance.*

*^§^ Comparison based on unpaired t-test or chi-square test.*

*^※^Groups of TUG time, heel strike angles, and stride speed by SDM tertile are shown in Supplementary Table 1.*

### Association of Cognitive Tests and Timed Up and Go

We examined the association between each cognitive test and TUG by multiple analyses, respectively, for two groups In the CI group, poor SDM performance reflecting memory deficit was associated with long TUG time (β, –0.51, 95% CI, –0.79 ∼ –0.22), while global cognitive function and other cognitive tests were not significantly related with TUG. For the CN group, all cognitive test was not associated with TUG (Supplementary Table 2).

### Association of Memory Deficit and Fall Risk

Table 2 shows multivariable regression models examined short delayed memory (SDM) and fall risk in different groups. In the CI group, compared to the relative excellent SDM performance (AVLT > 5), the elderly with bad SDM performance (AVLT ≤ 3) had higher fall risk (TUG time increased by 2.1 s, 95% CI, 0.54∼3.67; stride speed reduced by 0.09 m/s, 95% CI, −0.19 to –0.00; left heel strike angle reduced by 3.22°, 95% CI, −6.05 ∼−0.39; and right heel strike angle reduced by 2.37°, 95% CI, −5.09 to 0.36). The tendency was found in the association between immediate memory and fall risk, but without founding any association in the CN group (Table 2 and Supplementary Table 3).

**TABLE 2:** Association between short delayed memory (SDM) and fall risk in different groups^※^.

	Crude	Model I	Model II
	
		β (95%CI) P	
**CI group**			
**TUG time**			
AVLT-4 (continuous)	**−0.50 (−0.78, −0.23) 0.0008**	**−0.55 (−0.82, −0.27) 0.0003**	**−0.51 (−0.79, −0.22) 0.001**
**AVLT-4 (tertile)**			
High (*n* = 12)	0	0	0
Middle (*n* = 16)	0.92 (−0.79, 2.63) 0.296	0.77 (−0.93, 2.47) 0.377	1.21 (−0.52, 2.95) 0.179
Low (*n* = 20)	**1.99 (0.36, 3.63) 0.021**	**2.15 (0.55, 3.75) 0.011**	**2.10 (0.54, 3.67) 0.011**
Stride speed^▲^			
AVLT-4 (continuous)	0.01 (−0.01, 0.03) 0.200	0.02 (−0.00, 0.03) 0.072	0.02 (−0.00, 0.03) 0.083
**AVLT-4 (tertile)**			
High (*n* = 12)	0	0	0
Middle (*n* = 16)	−0.06 (−0.16, 0.04) 0.219	−0.03 (−0.13, 0.06) 0.506	−0.03 (−0.14, 0.07) 0.511
Low (*n* = 20)	**−0.09 (−0.18, 0.00) 0.060**	**−0.09 (−0.19, −0.00) 0.047**	**−0.09 (−0.19, −0.00) 0.051**
**Heel strike angles, left**			
AVLT-4 (continuous)	**0.49 (−0.03, 1.01) 0.071**	**0.61 (0.08, 1.15) 0.030**	**0.60 (0.03, 1.16) 0.044**
**AVLT-4 (tertile)**			
High (*n* = 12)	0	0	0
Middle (*n* = 16)	**−4.05 (−6.96, −1.15) 0.008**	**−3.60 (−6.64, −0.56) 0.025**	**−4.24 (−7.38, −1.09) 0.011**
Low (*n* = 20)	**−3.14 (−5.92, −0.36) 0.031**	**−3.29 (−6.14, −0.43) 0.029**	**−3.22 (−6.05, −0.39) 0.031**
**Heel strike angles, right**			
AVLT-4 (continuous)	**0.46 (−0.02, 0.95) 0.066**	**0.63 (0.14, 1.12) 0.015**	**0.60 (0.08, 1.11) 0.028**
AVLT-4 (tertile)			
High (*n* = 12)	0	0	0
Middle (*n* = 16)	−2.42 (−5.25, 0.41) 0.100	−2.09 (−5.02, 0.84) 0.169	−2.71 (−5.74, 0.32) 0.087
Low (*n* = 20)	−2.10 (−4.80, 0.60) 0.135	−2.43 (−5.19, 0.32) 0.090	−2.37 (−5.09, 0.36) 0.096
**CN group**			
**TUG time**			
AVLT-4 (continuous)	0.09 (−0.20, 0.37) 0.556	0.15 (−0.16, 0.46) 0.339	0.16 (−0.15, 0.47) 0.323
**AVLT-4 (tertile)**			
High (*n* = 40)	0	0	0
Middle (*n* = 13)	0.65 (−0.60, 1.90) 0.311	0.40 (−0.90, 1.69) 0.553	0.36 (−0.97, 1.68) 0.600
Low (*n* = 2)	−1.30 (−4.12, 1.52) 0.371	−0.85 (−3.77, 2.08) 0.572	−0.80 (−3.76, 2.16) 0.597
**Stride speed** ^▲^			
AVLT-4 (continuous)	0.01 (−0.01, 0.02) 0.466	0.00 (−0.02, 0.02) 0.859	0.00 (−0.02, 0.02) 0.890
**AVLT-4 (tertile)**			
High (*n* = 40)	0	0	0
Middle (*n* = 13)	−0.00 (−0.08, 0.08) 0.979	0.02 (−0.06, 0.10) 0.631	0.02 (−0.06, 0.11) 0.574
Low (*n* = 2)	0.08 (−0.10, 0.26) 0.384	0.09 (−0.10, 0.27) 0.351	0.08 (−0.10, 0.27) 0.379
**Heel strike angles, left**			
AVLT-4 (continuous)	0.31 (−0.23,0.86) 0.266	0.17 (−0.44,0.78) 0.589	0.15 (−0.46, 0.76) 0.630
**AVLT-4 (tertile)**			
High (*n* = 40)	0	0	0
Middle (*n* = 13)	−1.07 (−3.47, 1.33) 0.385	−0.78 (−3.26, 1.69) 0.537	−0.65 (−3.18, 1.87) 0.614
Low (*n* = 2)	2.20 (−3.25, 7.64) 0.432	3.67 (−1.92, 9.27) 0.204	3.53 (−2.11, 9.18) 0.226
**Heel strike angles, right**			
AVLT-4 (continuous)	−0.10 (−0.60, 0.41) 0.702	−0.26 (−0.80,0.29) 0.364	−0.27 (−0.82,0.29) 0.351
**AVLT-4 (tertile)**			
High (*n* = 40)	0	0	0
Middle (*n* = 13)	−0.06 (−2.26, 2.14) 0.957	0.20 (−2.03, 2.43) 0.859	0.27 (−2.02, 2.55) 0.820
Low (*n* = 2)	2.94 (−2.06, 7.94) 0.254	4.05 (−0.99, 9.09) 0.122	3.98 (−1.12, 9.09) 0.132

*Model I adjusts for age, sex, and education; Model II adjusts for age, sex, education, BMI, and HAMD.*

*^▲^Mean of left and right. Bold fonts indicate they had statistical significance.*

*^※^Association between immediate memory with fall risk is shown in Supplementary Table 2.*

### Association of Brain Volumes and Short Delayed Memory

Table 3 reports the relationship between brain volumes and SDM. In the CI group, small volumes of the hippocampus, temporal lobe, frontal lobe, parietal lobe, and serious medial temporal atrophy were associated with poor SDM performance after adjusting for covariates. Some of these brain regions were associated with immediate memory (*p* < 0.05). In the CN group, small hippocampus volumes were related to poor SDM performance after adjusting (Table 3).

**TABLE 3:** Association between brain volumes and SDM in different groups^※^.

	Crude	Model I	Model II
	
		β (95%CI) *p*	
**CI group**			
Cerebellum	−0.57 (−1.69, 0.54) 0.319	−0.37 (−1.61, 0.86) 0.557	−0.45 (−1.77, 0.87) 0.509
Hippocampus	**24.42 (13.37, 35.47) < 0.0001**	**25.76 (14.64, 36.88) < 0.001**	**26.40 (13.76, 39.04) 0.0002**
Hippocampus left	**44.65 (23.11, 66.19) 0.0002**	**47.46 (25.76, 69.16) 0.0001**	**47.35 (23.16, 71.53) 0.0005**
Hippocampus right	**46.71 (25.01, 68.41) 0.0001**	**48.69 (26.74, 70.64) < 0.0001**	**50.83 (25.47, 76.18) 0.0004**
MTA	**−0.09 (−0.14, −0.03) 0.005**	**−0.11 (−0.17, −0.05) 0.0009**	**−0.13 (−0.20, −0.06) 0.001**
MTA left	**−0.07 (−0.13, −0.02) 0.013**	**−0.10 (−0.16, −0.04) 0.001**	**−0.12 (−0.19, −0.05) 0.001**
MTA right	**−0.08 (−0.14, −0.03) 0.005**	**−0.10 (−0.16, −0.04) 0.002**	**−0.11 (−0.18, −0.04) 0.003**
Temporal lobe left	**2.67 (0.59, 4.75) 0.015**	**3.06 (0.89, 5.23) 0.008**	**3.24 (0.66, 5.81) 0.018**
Temporal lobe right	1.26 (−0.63, 3.14) 0.198	2.13 (−0.03, 4.29) 0.059	2.19 (−0.41, 4.78) 0.106
Frontal lobe left	**1.32 (−0.50, 3.15) 0.161**	**2.44 (0.47, 4.41) 0.019**	**2.72 (0.55, 4.89) 0.019**
Frontal lobe right	**1.21 (−0.74, 3.15) 0.230**	**2.74 (0.50, 4.99) 0.021**	**3.43 (0.86, 6.00) 0.013**
Parietal lobe left	**2.61 (0.23, 4.99) 0.036**	**3.29 (0.83, 5.76) 0.012**	**4.15 (1.30, 6.99) 0.007**
Parietal lobe right	1.14 (−0.89, 3.17) 0.277	2.57 (0.16, 4.97) 0.042	2.78 (0.06, 5.51) 0.053
Occipital lobe left	1.85 (−0.60, 4.29) 0.145	1.86 (−0.88, 4.60) 0.190	1.83 (−1.16, 4.83) 0.238
Occipital lobe right	1.49 (−0.49, 3.47) 0.146	1.47 (−0.66, 3.60) 0.184	1.41 (−1.09, 3.90) 0.276
**CN group**			
Cerebellum	0.04 (−0.76, 0.84) 0.917	0.32 (−0.50, 1.14) 0.449	0.35 (−0.47, 1.17) 0.406
Hippocampus	2.60 (−10.44, 15.64) 0.697	9.84 (−3.64, 23.32) 0.159	13.15 (−0.31, 26.60) 0.061
Hippocampus left	**5.09 (−19.30, 29.47) 0.684**	**22.37 (−2.73, 47.48) 0.087**	**28.91 (3.82, 54.00) 0.028**
Hippocampus right	4.62 (−21.07, 30.32) 0.725	13.56 (−13.01, 40.12) 0.322	19.19 (−7.37, 45.74) 0.163
MTA	−0.00 (−0.06, 0.05) 0.896	−0.02 (−0.09, 0.04) 0.445	−0.04 (−0.10, 0.02) 0.198
MTA left	−0.01 (−0.06, 0.05) 0.848	−0.04 (−0.10, 0.03) 0.247	−0.05 (−0.12, 0.01) 0.102
MTA right	−0.00 (−0.05, 0.05) 0.947	−0.01 (−0.06, 0.04) 0.708	−0.02 (−0.08, 0.03) 0.390
Temporal lobe left	0.60 (−1.51, 2.71) 0.580	1.09 (−1.08, 3.26) 0.328	1.40 (−0.86, 3.66) 0.230
Temporal lobe right	−0.04 (−2.28, 2.21) 0.973	0.49 (−1.72, 2.70) 0.664	0.26 (−2.01, 2.53) 0.824
Frontal lobe left	−0.01 (−1.33, 1.32) 0.993	0.50 (−0.84, 1.83) 0.469	0.69 (−0.70, 2.08) 0.336
Frontal lobe right	−0.64 (−1.94, 0.66) 0.341	−0.10 (−1.51, 1.31) 0.890	0.02 (−1.40, 1.45) 0.973
Parietal lobe left	−1.36 (−3.47, 0.75) 0.212	−0.40 (−2.59, 1.79) 0.720	0.24 (−2.04, 2.52) 0.835
Parietal lobe right	−1.91 (−3.70, −0.11) 0.042	−1.09 (−3.14, 0.97) 0.305	−0.95 (−3.02, 1.13) 0.376
Occipital lobe left	−0.71 (−3.00, 1.58) 0.548	−0.76 (−3.01, 1.48) 0.507	−0.54 (−2.78, 1.70) 0.640
Occipital lobe right	0.70 (−1.39, 2.78) 0.515	0.31 (−1.80, 2.43) 0.772	0.33 (−1.85, 2.52) 0.765

*Model I adjusts for age and sex; Model II adjusts for age, sex, BMI, hypertensive history, and diabetes history.*

*Bold fonts indicate they had statistical significance.*

*^※^Association between brain volumes and immediate memory is shown in Supplementary Table 3.*

### Association of Brain Volumes and Fall Risk

In the CI group, serious medial temporal atrophy (MTA), small volumes of the frontal lobe and occipital lobe were associated with long TUG time and small heel strike angles; small volumes of the temporal lobe, frontal lobe, and parietal lobe were associated with slow stride speed. But not found the relationships in CN elderly (Table 4 and Supplementary Tables 4–7).

**TABLE 4:** Association between brain volumes and timed up and go (TUG) time in different groups^※^.

	Crude	Model I	Model II
	
		β (95%CI) P	
**CI group**			
Cerebellum	0.06 (−1.16, 1.28) 0.921	0.10 (−1.18, 1.39) 0.873	−0.39 (−1.76, 0.98) 0.582
Hippocampus	**−25.00 (−37.17, −12.82) 0.0002**	**−24.78 (−37.11, −12.45) 0.0003**	**−22.75 (−36.19, −9.30) 0.002**
Hippocampus left	**−45.57 (−69.27, −21.87) 0.0005**	**−45.46 (−69.46, −21.45) 0.0006**	**−40.66 (−66.23, −15.08) 0.003**
Hippocampus right	**−47.62 (−71.54, −23.70) 0.0003**	**−47.01 (−71.21, −22.81) 0.0004**	**−43.75 (−70.53, −16.97) 0.002**
MTA	**0.13 (0.08, 0.19) < 0.0001**	**0.13 (0.08, 0.19) < 0.0001**	**0.14 (0.06, 0.22) 0.001**
MTA left	**0.11 (0.06, 0.17) 0.0002**	**0.12 (0.06, 0.17) 0.0002**	**0.11 (0.04, 0.18) 0.005**
MTA right	**0.13 (0.07, 0.18) < 0.0001**	**0.12 (0.07, 0.18) < 0.0001**	**0.13 (0.05, 0.20) 0.002**
Temporal lobe left	**−3.75 (−5.89, −1.61) 0.001**	**−3.80 (−5.96, −1.64) 0.001**	**−3.50 (−5.94, −1.05) 0.007**
Temporal lobe right	**−3.04 (−4.92, −1.16) 0.002**	**−3.10 (−5.07, −1.13) 0.003**	**−2.69 (−4.93, −0.46) 0.023**
Frontal lobe left	−2.18 (−4.09, −0.27) 0.030	−2.13 (−4.22, −0.05) 0.051	−1.89 (−4.15, 0.38) 0.111
Frontal lobe right	**−3.23 (−5.15, −1.32) 0.001**	**−3.56 (−5.70, −1.41) 0.002**	**−3.44 (−5.95, −0.93) 0.010**
Parietal lobe left	**−4.80 (−7.12, −2.49) 0.0002**	**−4.77 (−7.17, −2.37) 0.0003**	**−4.24 (−6.91, −1.56) 0.003**
Parietal lobe right	**−4.05 (−5.93, −2.16) 0.0001**	**−4.48 (−6.57, −2.39) 0.0001**	**−4.03 (−6.54, −1.51) 0.003**
Occipital lobe left	**−3.52 (−6.02, −1.01) 0.008**	**−3.52 (−6.04, −1.00) 0.008**	**−2.69 (−5.43, 0.06) 0.062**
Occipital lobe right	−2.28 (−4.37, −0.19) 0.038	−2.34 (−4.44, −0.23) 0.034	−1.56 (−3.99, 0.86) 0.214
**CN group**			
Cerebellum	0.45 (−0.40, 1.30) 0.304	0.48 (−0.42, 1.38) 0.298	0.52 (−0.44, 1.48) 0.292
Hippocampus	4.90 (−9.03, 18.84) 0.493	5.16 (−9.25, 19.58) 0.485	5.22 (−9.82, 20.26) 0.499
Hippocampus left	7.68 (−18.57, 33.93) 0.568	7.69 (−19.80, 35.19) 0.585	7.21 (−21.36, 35.77) 0.623
Hippocampus right	10.12 (−17.21, 37.45) 0.471	10.98 (−16.84, 38.80) 0.442	11.60 (−17.43, 40.63) 0.437
MTA	0.00 (−0.06, 0.06) 0.964	0.02 (−0.04, 0.09) 0.496	0.02 (−0.06, 0.09) 0.675
MTA left	−0.02 (−0.08, 0.05) 0.616	0.01 (−0.06, 0.08) 0.830	−0.00 (−0.08, 0.07) 0.989
MTA right	0.01 (−0.04, 0.07) 0.616	0.03 (−0.03, 0.08) 0.332	0.02 (−0.04, 0.08) 0.478
Temporal lobe left	1.76 (−0.45, 3.98) 0.125	1.31 (−1.04, 3.65) 0.280	1.66 (−0.77, 4.08) 0.187
Temporal lobe right	1.15 (−1.24, 3.54) 0.350	0.79 (−1.65, 3.23) 0.530	0.93 (−1.63, 3.50) 0.480
Frontal lobe left	0.36 (−1.06, 1.78) 0.622	−0.04 (−1.53, 1.46) 0.961	0.20 (−1.39, 1.79) 0.803
Frontal lobe right	0.30 (−1.11, 1.71) 0.678	−0.14 (−1.66, 1.37) 0.853	−0.03 (−1.62, 1.55) 0.968
Parietal lobe left	−0.19 (−2.55, 2.16) 0.872	−0.87 (−3.37, 1.62) 0.494	−0.78 (−3.41, 1.85) 0.563
Parietal lobe right	0.31 (−1.72, 2.34) 0.766	−0.47 (−2.80, 1.87) 0.697	−0.56 (−2.96, 1.84) 0.649
Occipital lobe left	0.10 (−2.37, 2.58) 0.936	−0.06 (−2.52, 2.41) 0.963	0.03 (−2.61, 2.67) 0.981
Occipital lobe right	0.63 (−1.63, 2.88) 0.587	0.52 (−1.75, 2.78) 0.656	0.80 (−1.58, 3.17) 0.514

*Model I adjusts for age and sex; Model II adjusts for age, sex, BMI, hypertensive history, and diabetes history.*

*^※^Association between brain volumes and stride speed, left heel strike angle, and right heel strike angle is shown in Supplementary Tables 4–6. Bold fonts indicate they had statistical significance.*

### Sensitivity Analyses

In the CI group, when modeling SDM as a continuous variable, poor SDM was associated with increased TUG time, reduced heel strike angle, and slow stride speed after adjusting for covariates. When modeling SDM as tripartite variables, poor SDM was associated with increased TUG time, reduced heel strike angle, and slow stride speed (Table 2).

## Discussion

This study evaluated the relationship between memory deficit and high fall risk in aMCI and mild AD elderly. Moreover, we explored its underlying neuroanatomical linkage. Our results suggested that memory deficit was associated with increased fall risk, which was reflected by long TUG time, small heel strike angles, and slow stride speed in the elderly with aMCI and mild AD. Furthermore, the association might be mediated by the medial temporal, frontal, and parietal lobes.

This study suggests that poor memory, especially short delayed memory, was associated with increased fall risk among people with aMCI and mild AD. Several previous evidence indicated that different cognitive domains related fall risk among the elderly. Beauchet et al. (2014) suggested that a high risk of falls was associated with declined memory in the community-dwellers without dementia. Inconsistently, poor visuospatial performance was associated with increased fall risk among MCI and AD patients (Ansai et al., 2017). In these studies, the cognitive domains were assessed by sub-scores of Adden Brooke Cognitive Examination-Revised Visual or Short Mini-Mental State Examination, brief screening tools of global cognitive function. Given the controversial results, further exploring the linkage between cognitive domains and fall risk is necessary. In our study, a comprehensive neuropsychology battery cognitive task, which was more accurate to reflect the severity of cognitive impairment, was applied to evaluate the cognitive function of a given domain.

Moreover, unlike previous studies, inertial-sensor-based wearable devices were used to evaluate the kinetic parameters in this study. It was possible to assess fall risk efficiently by several parameters: TUG time, stride speed, and heel strike angles. Therefore, the results of this study, which was memory deficit associated with high fall risk in individuals with aMCI and mild AD, were more reliable.

The association of memory deficit and high fall risk might be partly explained by the relationship of brain volumes with memory and fall risk. According to our analysis, in the cognitive impairment elderly, medial temporal, frontal, and parietal lobes atrophy was associated with poor short delayed memory performance and high fall risk (i.e., long TUG time, slow stride speed, and small heel strike angles). However, there was no association found in cognitively normal elderly. Thus, medial temporal, frontal, and parietal lobes atrophy might play an essential role in mediating the association between memory deficit and high fall risk.

There was no association between regional brain volumes and high fall risk among healthy adults in this study. In healthy elderly, adaptive motor behaviors are initiated essentially by corticospinal volleys in the motor neurons. Then, corticofugal neurons received the graded excitatory and inhibitory information from different afferent pathways. Next, the balances among these synaptic impingements were encoded in the final pattern of impulses discharging from the motor cortex (Marchiafava, 1968). According to this theory, the brain’s default mode network consisted of bilateral cortical areas, including parietal, prefrontal, medial and lateral temporal cortices (Raichle, 2015), which might help to explain our negative results in cognitively normal elderly. In addition, it has been suggested that the default mode network of healthy adults was more activated in contrast to MCI and AD patients (Rombouts et al., 2005). Therefore, given the relative normal function of motion and cognition (i.e., regular default mode network), there might be no significant association between regional brain atrophy and high fall risk in cognitively normal adults.

Our results also confirmed that reduced volume of various brain regions, including the medial temporal frontal and parietal lobes, were associated with high fall risk in aMCI and mild AD elderly. Recently, two studies investigated the linkage between regional brain volume and TUG time in MCI. Allali et al. (2020) suggested that volumetric reduction in bilateral cerebellar was associated with increased TUG time among individuals with MCI. In this study, analysis was not conducted for the subgroup of aMCI. Another research reported that low hippocampal volume was associated with TUG time among 66 subjects with naMCI. This study also analyzed a subset of 25 aMCI subjects and found that specific regional atrophy and TUG did not show significant association, which might be due to the relatively small sample size (Allali et al., 2016). Thus, the underlying neuropathological mechanism regarding the specific regional brain atrophy related to fall risk remains uncertain among aMCI. Exploring the relationship between brain structural volume and fall risk further in aMCI is essential.

It is well-known that aMCI was a prodromal stage of AD. Mild AD, the development phase of aMCI, was the early AD. Patients with mild AD had a mild cognitive decline and limited functional impairment. Besides, the prevalence of falls in patients with MCI and mild AD was similar (52.6% and 51.4%) (Ansai et al., 2017). Given these, we combined the aMCI and mild AD patients to explore the relationship between brain volume and fall risk. Therefore, our results might first reveal the relationship between brain regional volume and fall risk by different parameters in the elderly with aMCI and mild AD. Furthermore, this study found that several small volumes of brain regions, including the hippocampus, medial temporal lobe, temporal lobe, frontal lobe, parietal lobe, and occipital lobe, were associated with TUG time in aMCI and mild AD. The finding might be explained by the default mode network as well.

To date, few researchers attempted to investigate the underlying neuropathology mechanism of which motor dysfunction is closely related to cognition in aMCI and mild AD patients. In this field, several studies have explored the mechanism in healthy adults. One functional magnetic resonance imaging (fMRI) study reported that the superior parietal lobe plays a crucial role in cognitive-motor dual tasks among healthy elderly (Bürki et al., 2017). Bürki et al. (2017) reported that the brain regions, from inferior frontal to superior, middle temporal gyrus, were significantly activated in cognitive-motor dual tasks in 12 healthy young adults using near-infrared spectroscopy (fNIRS) (Metzger et al., 2017). In light of these findings, brain regions such as the parietal lobe, frontal lobe, and temporal lobe might play a distinct and crucial role in regulating cognition and motor function in healthy adults. Compared to functional imaging, the structure image of MRI was a more routine imaging method in clinical practice. By volumetric MRI, we found that medial temporal lobe atrophy was simultaneously associated with poor memory, long TUG time, small heel stride angles, and stride speed in aMCI and mild AD patients. The results were in line with recent functional imaging studies in healthy adults. Therefore, we confirmed that the medial temporal, frontal, and parietal lobes atrophy, might play an essential role in mediating the association between memory deficit and high fall risk in cognitive impairment patients.

Overall, our results suggested that memory deficit was associated with high fall risk. Meanwhile, they were related to medial temporal, frontal, and parietal lobes atrophy among aMCI and mild AD. Hence, increased fall risk, assessed by TUG time, heel stride angles, and stride speed might be significant potential kinematics markers to reflect early memory impairment. These findings have important implications for early discernment of memory deficit of the elderly by kinematics parameters and for the prevention of falls. As posture and balance could be intervened with training, improved motor function by training might prevent falls and delay the progression of memory decline. Additionally, assessed by wearable instruments, TUG time, heel stride angles, and stride speed might be objective and convenient parameters for dynamic monitoring of both memory and fall risk.

Several limitations should be acknowledged. First, this study is a cross-sectional design with its inherent deficiency. A follow-up examination is necessary for revealing the association of high fall risk and memory deficits among aMCI and mild AD in depth. Second, we collected kinetic parameters in single-task gait assessment rather than in dual-task, which was more sensitive for discriminating between MCI and healthy elderly. In future research, a dual-task gait assessment will be preferred to better distinguish between aMCI and mild AD. Third, gait control requires a complex sensorimotor function. It is controlled by integrated cortical, subcortical, and spinal networks. As cortex, the subcortex also plays a vital role in gait control. However, there was no information about subcortical lesion markers in this study, which will be a concern in the future. Lastly, due to the relatively small sample size (*n* = 11), the analysis efficiency is insufficient to examine the association of cognitive function and fall risk for the mild AD group. Therefore, we combined the aMCI and patients with mild AD as our data did not support analysis for aMCI and AD groups, respectively.

## Conclusion

Our results suggested that memory deficit was associated with high fall risk in the elderly with aMCI and mild AD. The medial temporal, frontal, and parietal lobes atrophy might mediate the association. Additionally, an increased fall risk, tested by TUG time, heel stride angles, and stride speed, might be objective and convenient kinematics markers for dynamic monitoring of both memory and fall risk.

## Data Availability Statement

The raw data supporting the conclusions of this article will be made available by the authors, without undue reservation.

## Ethics Statement

The studies involving human participants were reviewed and approved by the Research Ethics Board of the First People’s Hospital of Foshan. The patients/participants provided their written informed consent to participate in this study.

## Author Contributions

SH, HX, and SP contributed to the conception of the study and design, study supervision, interpretation of data, and manuscript preparation. XZ contributed to imaging techniques and all examinations. CZ, YW, LC, ZL, and ST helped to perform the analysis and critical revision of the manuscript. YJL, YF, PS, WW, and JL contributed to acquiring imaging data and kinetic parameters. WZ, QH, and BL contributed to purchasing neuropsychological evaluation. LS, YSL, and VM contributed to designing the MRI data analysis solutions. All authors contributed to the article and approved the submitted version.

## Conflict of Interest

LS was the director of the BrainNow Medical Technology Ltd. YSL was employed by the BrainNow Medical Technology Ltd. VM was the Chief Medical Advisor of BrainNow Medical Technology Ltd. The remaining authors declare that the research was conducted in the absence of any commercial or financial relationships that could be construed as a potential conflict of interest.

## Publisher’s Note

All claims expressed in this article are solely those of the authors and do not necessarily represent those of their affiliated organizations, or those of the publisher, the editors and the reviewers. Any product that may be evaluated in this article, or claim that may be made by its manufacturer, is not guaranteed or endorsed by the publisher.

## Footnotes

1
http://www.R-project.org

2
www.empowerstats.com
