# Supplementary material for: High Fall Risk Associated With Memory Deficit and Brain Lobes Atrophy Among Elderly With Amnestic Mild Cognitive Impairment and Mild Alzheimer’s Disease
Source: Front Neurosci. 2022 Jun 8;16:896437. doi: 10.3389/fnins.2022.896437 (PMC9213689; doi:10.3389/fnins.2022.896437)
Supplement: Supplementary file 1 [file Data_Sheet_1.PDF]

Supplementary Table 1. Gait Parameters Characteristics of Participants

	Total(N=103)	Group CI (N=48)	Group CN (N=55)	P[§]
**TUG time (continuous) , s**	10.90 ± 2.23	11.44 ± 2.38	10.41 ± 1.99	0.018
Groups of TUG time by AVLT-4 tertile,				
High	10.30 ± 2.00	**10.31 ± 2.03**	10.30 ± 2.02	
Middle	11.10 ± 2.03	**11.23 ± 2.16**	10.95 ± 1.92	
Low	12.00 ± 2.60	**12.30 ± 2.51**	9.00 ± 1.41	
**Heel strike angles, degrees**				
**Left (continuous)**	30.93 ±3.70	30.23 ±3.83	31.55 ±3.50	0.069
Groups of left heel strike angles by AVLT-4 tertile				
High	31.97 ± 3.88	**33.51 ± 2.33**	31.50 ± 4.15	
Middle	29.89 ± 3.61	**29.46 ± 4.20**	30.43 ± 2.80	
Low	30.67 ± 4.22	**30.37 ± 4.31**	33.70 ± 0.14	
**Right (continuous)**	31.11 ± 3.95	30.85 ± 4.12	31.33 ± 3.82	0.542
Groups of right heel strike angles by AVLT-4 tertile				
High	31.56 ± 3.47	31.91 ± 2.95	31.46 ± 3.63	
Middle	30.34 ± 3.30	29.49 ± 3.19	31.40 ± 3.24	
Low	30.23 ± 4.55	29.81 ± 4.55	34.40 ± 1.84	
**Stride speed,m/s**				
**Left (continuous)**	0.90 ± 0.13	0.89±0.13	0.91 ± 0.12	0.099
Groups of left stride speed by AVLT-4 tertile				
High	0.92 ± 0.14	0.95 ± 0.18	0.91 ± 0.13	
Middle	0.90 ± 0.11	0.89 ± 0.10	0.91 ± 0.12	
Low	0.87 ± 0.12	0.86 ± 0.12	0.99 ± 0.06	
**Right (continuous)**	0.90 ± 0.13	0.89± 0.13	0.91 ±0.12	0.099
Groups of right stride speed by AVLT-4 tertile				
High	0.92 ± 0.14	0.95 ± 0.18	0.91 ± 0.13	
Middle	0.90 ± 0.11	0.89 ± 0.10	0.91 ± 0.12	
Low	0.87 ± 0.12	0.86 ± 0.12	0.99 ± 0.06	
**Cadence, steps/min**				
**Left (continuous)**	98.82 ± 8.12	98.52 ±8.38	99.07 ± 7.95	0.820
Groups of left cadence by AVLT-4 tertile				
High	98.90 ± 8.76	99.29 ± 10.72	98.78 ± 8.23	
Middle	99.74 ± 7.64	99.33 ± 7.72	100.25 ± 7.81	
Low	97.40 ± 7.28	97.42 ± 7.61	97.22 ± 3.34	
**Right (continuous)**	98.82 ± 8.12	98.52 ± 8.38	99.07 ± 7.95	0.820
Groups of right cadence by AVLT-4 tertile				
High	98.90 ± 8.76	99.29 ± 10.72	98.78 ± 8.23	
Middle	99.74 ± 7.64	99.33 ± 7.72	100.25 ± 7.81	
Low	97.40 ± 7.28	97.42 ± 7.61	97.22 ± 3.34	

§ Comparison based on unpaired t-test or chi-square test. Bold fonts indicate they had statistical significance.

Supplementary Table 2 Association of Cognitive function with TUG

	Crude	Model I	Model II
		β (95%CI) P	
**Group CN**			
MMSE	-0.06 (-0.36, 0.23) 0.672	0.00 (-0.35, 0.35) 0.993	-0.07 (-0.45, 0.31) 0.717
Immediate memory	0.29 (-0.03, 0.62) 0.081	0.35 (-0.01, 0.72) 0.066	0.69 (-0.81, 2.19) 0.374
SDM	0.09 (-0.20, 0.37) 0.556	0.15 (-0.16, 0.46) 0.339	0.16 (-0.15, 0.47) 0.323
LDM	0.22 (-0.01, 0.45) 0.061	0.33 (0.08, 0.58) 0.013	0.26 (0.01, 0.50) 0.044
Executive Function	-0.03 (-0.09, 0.03) 0.335	-0.03 (-0.09, 0.03) 0.334	-0.03 (-0.11, 0.04) 0.394
Attention	0.03 (-0.03, 0.10) 0.310	0.04 (-0.03, 0.12) 0.256	-0.01 (-0.10, 0.08) 0.802
Visual Spatial Ability	0.28 (-0.10, 0.66) 0.153	0.32 (-0.09, 0.73) 0.136	0.25 (-0.27, 0.77) 0.355
Language	-0.01 (-0.21, 0.18) 0.882	0.09 (-0.19, 0.36) 0.540	-0.11 (-0.40, 0.19) 0.483
**Group CI**			
MMSE	-0.17 (-0.36, 0.02) 0.094	-0.22 (-0.44, -0.00) 0.055	-0.15 (-0.36, 0.07) 0.194
Immediate memory	-0.64 (-1.17, -0.11) 0.022	-0.63 (-1.19, -0.07) 0.034	-0.55 (-1.11, 0.02) 0.064
**SDM**	**-0.50 (-0.78, -0.23) 0.001**	**-0.55 (-0.83, -0.27) 0.001**	**-0.51 (-0.79, -0.22) 0.001**
LDM	-0.33 (-0.63, -0.03) 0.035	-0.34 (-0.67, -0.01) 0.050	-0.28 (-0.58, 0.03) 0.083
Executive Function	0.03 (0.01, 0.06) 0.021	0.03 (0.00, 0.06) 0.038	0.02 (-0.01, 0.05) 0.126
Attention	-0.07 (-0.13, -0.00) 0.042	-0.07 (-0.14, 0.01) 0.084	-0.05 (-0.12, 0.02) 0.161
Visual Spatial Ability	-0.05 (-0.31, 0.21) 0.733	-0.15 (-0.47, 0.17) 0.372	-0.15 (-0.47, 0.16) 0.353
Language	-0.08 (-0.23, 0.06) 0.246	-0.15 (-0.33, 0.04) 0.131	-0.13 (-0.31, 0.05) 0.180

Model I adjust for age; sex; education; Model II adjust for age; sex; education; BMI; HAMD scores.

Supplementary Table 3. Association between Immediate Memory (IM) and Fall Risk

	Crude	Model I	Model II
		β (95%CI) P	
**CI group**			
**TUG time**			
**AVLT-1 (continuous)**	**-0.64 (-1.17, -0.11) 0.022**	**-0.63 (-1.19, -0.07) 0.034**	**-0.55 (-1.11, 0.02) 0.064**
**AVLT-1 (tertile)**			
High (n=27)	0	0	0
Low (n=21)	1.18 (-0.14, 2.51) 0.087	1.12 (-0.23, 2.48) 0.110	0.95 (-0.39, 2.29) 0.171
**Stride speed**			
**AVLT-1 (continuous)**	0.00 (-0.03, 0.04) 0.804	0.02 (-0.02, 0.05) 0.343	0.02 (-0.02, 0.05) 0.380
**AVLT-1 (tertile)**			
High (n=27)	0	0	0
Low (n=21)	-0.01 (-0.08, 0.07) 0.856	-0.03 (-0.10, 0.05) 0.477	-0.03 (-0.10, 0.05) 0.516
**Heel strike angles, left**			
**AVLT-1 (continuous)**	0.26 (-0.71,1.23) 0.596	0.50 (-0.54,1.53) 0.354	0.43 (-0.63, 1.49) 0.430
**AVLT-1 (tertile)**			
High (n=27)	0	0	0
Low (n=21)	-0.48 (-2.85, 1.89) 0.694	-0.97 (-3.42, 1.47) 0.440	-0.84 (-3.33, 1.65) 0.512
**Heel strike angles, right**			
**AVLT-1 (continuous)**	0.05 (-0.86, 0.95) 0.917	0.29 (-0.68,1.26) 0.561	0.19 (-0.79,1.18) 0.701
**AVLT-1 (tertile)**			
High (n=27)	0	0	0
Low (n=21)	-0.06 (-2.26, 2.15) 0.960	-0.46 (-2.75, 1.82) 0.692	-0.27 (-2.58, 2.04) 0.818
**CN group**			
**TUG time**			
**AVLT-1 (continuous)**	0.29 (-0.03, 0.62) 0.081	0.35 (-0.01, 0.72) 0.066	0.69 (-0.81, 2.19) 0.374
**AVLT-1 (tertile)**			
High (n=45)	0	0	0
Low (n=10)	-0.91 (-2.27, 0.45) 0.194	-0.74 (-2.20, 0.72) 0.325	-0.69 (-2.19, 0.81) 0.374
**Stride speed**▲			
**AVLT-1 (continuous)**	0.01 (-0.01, 0.04) 0.165	0.01 (-0.01, 0.04) 0.250	0.01 (-0.01, 0.04) 0.241
**AVLT-1 (tertile)**			
High (n=45)	0	0	0
Low (n=10)	0.01 (-0.08, 0.10) 0.822	0.02 (-0.08, 0.11) 0.729	0.01 (-0.08, 0.11) 0.814
**Heel strike angles, left**			
**AVLT-1 (continuous)**	0.24 (-0.40,0.88) 0.471	-0.04 (-0.77,0.70) 0.923	-0.02 (-0.76,0.72) 0.955
**AVLT-1 (tertile)**			
High (n=45)	0	0	0
Low (n=10)	0.03 (-2.62, 2.67) 0.984	0.87 (-1.98, 3.73) 0.552	0.65 (-2.28, 3.58) 0.664
**Heel strike angles, right**			
**AVLT-1 (continuous)**	0.05 (-0.54, 0.64) 0.861	-0.18 (-0.84, 0.49) 0.607	-0.17 (-0.84,0.50) 0.625
**AVLT-1 (tertile)**			
High (n=45)	0	0	0
Low (n=10)	0.56 (-1.86, 2.97) 0.652	1.29 (-1.27, 3.85) 0.327	1.22 (-1.42, 3.86) 0.368

Model I adjusts for age, sex, and education; Model II adjusts for age, sex, education, BMI and HAMD.
▲ Mean of left and right. Bold fonts indicate they had statistical significance.

Supplementary Table 4. Association between Brain Volumes and Immediate Memory

	Crude	Model I	Model II
	β (95%CI) P		
**CI group**			
Cerebellum	-0.07 (-0.70, 0.56) 0.830	0.13 (-0.53, 0.79) 0.704	0.10 (-0.59, 0.80) 0.770
Hippocampus	7.03 (0.04, 14.01) 0.054	7.23 (0.32, 14.15) 0.046	6.55 (-1.20, 14.30) 0.106
Hippocampus left	12.12 (-1.36,25.61) 0.084	12.95 (-0.38, 26.28) 0.063	11.56 (-3.04, 26.16) 0.129
Hippocampus right	14.05 (0.46, 27.64) 0.048	13.97 (0.51, 27.44) 0.048	12.71 (-2.67, 28.08) 0.113
**MTA**	**-0.03 (-0.06, 0.01) 0.124**	**-0.04 (-0.08, -0.01) 0.019**	**-0.05 (-0.09, -0.01) 0.020**
**MTA left**	**-0.02 (-0.05, 0.01) 0.176**	**-0.04 (-0.07, -0.00) 0.035**	**-0.04 (-0.08, -0.01) 0.025**
**MTA right**	**-0.03 (-0.06, 0.01) 0.120**	**-0.04 (-0.07, -0.01) 0.022**	**-0.04 (-0.08, -0.00) 0.034**
**Temporal lobe left**	**0.87 (-0.33, 2.08) 0.162**	**1.30 (0.10, 2.49) 0.039**	**1.41 (0.02, 2.80) 0.053**
Temporal lobe right	0.16 (-0.90, 1.22) 0.770	0.86 (-0.31, 2.03) 0.157	0.97 (-0.41, 2.35) 0.175
Frontal lobe left	0.17 (-0.86, 1.20) 0.749	0.50 (-0.61, 1.62) 0.378	0.63 (-0.58, 1.85) 0.313
Frontal lobe right	-0.12 (-1.22, 0.97) 0.828	0.58 (-0.69, 1.84) 0.374	0.78 (-0.67, 2.23) 0.299
**Parietal lobe left**	**1.28 (-0.06, 2.61) 0.066**	**1.72 (0.40, 3.03) 0.014**	**2.22 (0.73, 3.71) 0.005**
**Parietal lobe right**	**0.60 (-0.52, 1.73) 0.300**	**1.55 (0.28, 2.81) 0.021**	**1.69 (0.29, 3.10) 0.023**
Occipital lobe left	0.18 (-1.21, 1.56) 0.802	0.19 (-1.30, 1.68) 0.801	0.06 (-1.54, 1.67) 0.939
Occipital lobe right	0.59 (-0.52, 1.70) 0.305	0.82 (-0.31, 1.95) 0.163	0.68 (-0.63, 2.00) 0.314
**CN group**			
Cerebellum	-0.16 (-0.85, 0.52) 0.640	-0.10 (-0.77, 0.57) 0.777	-0.04 (-0.72, 0.65) 0.919
Hippocampus	-0.59(-11.81,10.63) 0.918	2.96 (-8.16, 14.08) 0.604	4.40 (-7.13, 15.94) 0.458
Hippocampus left	-5.05(-25.99,15.89) 0.638	5.48 (-15.44, 26.40) 0.610	7.94 (-13.89, 29.78) 0.479
Hippocampus right	3.21 (-18.87,25.30) 0.776	5.29 (-16.41, 26.99) 0.634	8.12 (-14.27, 30.50) 0.480
MTA	-0.00 (-0.05, 0.05) 0.939	-0.02 (-0.07, 0.03) 0.543	-0.01 (-0.07, 0.04) 0.593
MTA left	0.00 (-0.05, 0.06) 0.848	-0.02 (-0.07, 0.04) 0.565	-0.01 (-0.07, 0.04) 0.598
MTA right	-0.01 (-0.05, 0.04) 0.774	-0.01 (-0.05, 0.03) 0.573	-0.01 (-0.06, 0.03) 0.638
Temporal lobe left	1.79 (0.06, 3.52) 0.047	2.35 (0.71, 3.99) 0.007	2.30 (0.53, 4.07) 0.014
Temporal lobe right	0.57 (-1.33, 2.46) 0.561	1.12 (-0.65, 2.88) 0.221	0.87 (-0.99, 2.73) 0.362
Frontal lobe left	0.46 (-0.66, 1.58) 0.423	0.89 (-0.17, 1.94) 0.106	0.78 (-0.36, 1.92) 0.187
Frontal lobe right	-0.03 (-1.14, 1.09) 0.960	0.54 (-0.59, 1.66) 0.353	0.45 (-0.72, 1.62) 0.456
Parietal lobe left	-0.58 (-2.38, 1.23) 0.535	0.32 (-1.44, 2.09) 0.721	0.52 (-1.36, 2.40) 0.590
Parietal lobe right	-1.26 (-2.81, 0.28) 0.116	-0.56 (-2.24, 1.11) 0.512	-0.44 (-2.16, 1.29) 0.621
Occipital lobe left	-0.41 (-2.35, 1.54) 0.682	-0.64 (-2.45, 1.17) 0.492	-0.61 (-2.46, 1.24) 0.520
Occipital lobe right	0.70 (-1.07, 2.46) 0.442	0.01 (-1.70, 1.72) 0.988	-0.10 (-1.90, 1.71) 0.917

Model I adjusts for age, education, and sex; Model II adjusts for age, education, sex, HAMD, hypertensive history, and diabetes history. Bold fonts indicate they had statistical significance.

Supplementary Table 5. Association between Brain Volumes and Stride Speed

	Crude	Model I	Model II
		**β (95%CI) P**	
**CI group**			
Cerebellum	0.01 (-0.06, 0.08) 0.793	-0.01 (-0.08, 0.06) 0.820	0.01 (-0.06, 0.09) 0.730
Hippocampus	0.25 (-0.53, 1.04) 0.534	0.21 (-0.57, 0.99) 0.596	0.27 (-0.58, 1.13) 0.534
Hippocampus left	0.23 (-1.28, 1.74) 0.767	0.11 (-1.38, 1.60) 0.885	0.15 (-1.47, 1.76) 0.860
Hippocampus right	0.73 (-0.79, 2.25) 0.351	0.70 (-0.80, 2.20) 0.364	0.93 (-0.75, 2.61) 0.282
MTA	-0.00 (-0.01, 0.00) 0.078	-0.00 (-0.01, 0.00) 0.116	-0.00 (-0.01, 0.00) 0.317
MTA left	-0.00 (-0.01, 0.00) 0.113	-0.00 (-0.01, 0.00) 0.221	-0.00 (-0.01, 0.00) 0.568
MTA right	-0.00 (-0.01, 0.00) 0.083	-0.00 (-0.01, 0.00) 0.082	-0.00 (-0.01, 0.00) 0.200
Temporal lobe left	0.04 (-0.09, 0.17) 0.575	0.02 (-0.11, 0.16) 0.720	0.02 (-0.13, 0.18) 0.764
**Temporal lobe right**	**0.13 (0.02, 0.24) 0.028**	**0.11 (-0.00, 0.23) 0.057**	**0.12 (-0.01, 0.25) 0.090**
Frontal lobe left	0.08 (-0.03, 0.19) 0.153	0.07 (-0.04, 0.19) 0.218	0.07 (-0.07, 0.20) 0.331
**Frontal lobe right**	**0.18 (0.08, 0.29) 0.001**	**0.19 (0.07, 0.31) 0.002**	**0.20 (0.06, 0.34) 0.009**
Parietal lobe left	0.14 (-0.01, 0.28) 0.067	0.13 (-0.02, 0.28) 0.090	0.11 (-0.06, 0.27) 0.213
**Parietal lobe right**	**0.15 (0.03, 0.26) 0.016**	**0.15 (0.02, 0.27) 0.028**	**0.14 (-0.01, 0.29) 0.080**
Occipital lobe left	0.11 (-0.04, 0.26) 0.154	0.11 (-0.04, 0.25) 0.145	0.09 (-0.07, 0.25) 0.270
Occipital lobe right	0.06 (-0.06, 0.18) 0.302	0.06 (-0.05, 0.18) 0.291	0.07 (-0.07, 0.20) 0.358
**CN group**			
Cerebellum	-0.02 (-0.08, 0.03) 0.391	-0.01 (-0.07, 0.04) 0.658	-0.01 (-0.07, 0.05) 0.642
Hippocampus	-0.14 (-1.01, 0.73) 0.752	0.02 (-0.87, 0.91) 0.970	-0.11 (-1.04, 0.82) 0.819
Hippocampus left	-0.33 (-1.95, 1.29) 0.692	0.02 (-1.67, 1.70) 0.982	-0.20 (-1.96, 1.56) 0.826
Hippocampus right	-0.17 (-1.88, 1.55) 0.850	0.06 (-1.66, 1.78) 0.947	-0.18 (-1.98, 1.62) 0.844
MTA	0.00 (-0.00, 0.00) 0.693	-0.00 (-0.00, 0.00) 0.720	-0.00 (-0.00, 0.00) 0.858
MTA left	0.00 (-0.00, 0.00) 0.984	-0.00 (-0.01, 0.00) 0.274	-0.00 (-0.01, 0.00) 0.351
MTA right	0.00 (-0.00, 0.00) 0.507	0.00 (-0.00, 0.00) 0.818	0.00 (-0.00, 0.00) 0.684
Temporal lobe left	-0.00 (-0.14, 0.14) 0.990	0.05 (-0.10, 0.19) 0.533	0.05 (-0.10, 0.20) 0.528
Temporal lobe right	0.02 (-0.13, 0.17) 0.834	0.05 (-0.10, 0.20) 0.487	0.08 (-0.08, 0.24) 0.333
Frontal lobe left	0.01 (-0.08, 0.10) 0.836	0.04 (-0.05, 0.13) 0.383	0.05 (-0.05, 0.14) 0.344
Frontal lobe right	0.03 (-0.06, 0.11) 0.555	0.07 (-0.02, 0.16) 0.151	0.07 (-0.02, 0.17) 0.137
Parietal lobe left	0.03 (-0.11, 0.18) 0.650	0.09 (-0.06, 0.24) 0.240	0.07 (-0.09, 0.23) 0.389
Parietal lobe right	-0.01 (-0.13, 0.12) 0.917	0.07 (-0.07, 0.21) 0.317	0.06 (-0.08, 0.21) 0.403
Occipital lobe left	0.03 (-0.13, 0.18) 0.748	0.03 (-0.12, 0.18) 0.690	0.01 (-0.15, 0.18) 0.872
Occipital lobe right	-0.01 (-0.15, 0.13) 0.897	-0.01 (-0.15, 0.13) 0.858	-0.02 (-0.17, 0.13) 0.776

Model I adjusts for age, and sex; Model II adjusts for age, sex, BMI, hypertensive history, and diabetes history.

Bold fonts indicate they had statistical significance.

Supplementary Table 6. Association between Brain Volumes and Left Heel Strike Angle

	Crude	Model I	Model II
		β (95%CI) P	
		**CI group**	
Cerebellum	0.88 (-1.22, 2.98) 0.415	1.31 (-0.86, 3.49) 0.242	0.82 (-1.64, 3.28) 0.516
Hippocampus	-8.78 (-33.16, 15.61) 0.484	-8.16 (-32.73, 16.40) 0.518	-5.87 (-33.08, 21.34) 0.674
Hippocampus left	-22.05 (-68.56, 24.47) 0.357	-20.04 (-67.02, 26.95) 0.407	-16.53 (-67.47, 34.40) 0.528
Hippocampus right	-11.04 (-58.71, 36.64) 0.652	-10.83 (-58.76, 37.10) 0.660	-5.28 (-59.17, 48.61) 0.848
**MTA**	**0.14 (0.03, 0.25) 0.014**	**0.14 (0.03, 0.26) 0.016**	**0.15 (-0.01, 0.30) 0.074**
**MTA left**	**0.11 (0.01, 0.22) 0.035**	**0.11 (0.00, 0.22) 0.052**	**0.10 (-0.04, 0.24) 0.185**
**MTA right**	**0.14 (0.03, 0.24) 0.012**	**0.14 (0.04, 0.25) 0.010**	**0.15 (0.00, 0.30) 0.050**
Temporal lobe left	-1.53 (-5.66, 2.59) 0.470	-1.25 (-5.43, 2.92) 0.559	-0.98 (-5.77, 3.81) 0.689
Temporal lobe right	-2.49 (-6.01, 1.03) 0.172	-2.28 (-5.95, 1.40) 0.230	-1.76 (-6.00, 2.49) 0.421
Frontal lobe left	-2.82 (-7.42, 1.77) 0.234	-1.79 (-5.51, 1.92) 0.349	-1.54 (-5.71, 2.63) 0.474
**Frontal lobe right**	**-3.58 (-7.28, 0.12) 0.064**	**-5.98 (-9.70, -2.27) 0.002**	**-6.32 (-10.80, -1.84) 0.008**
Parietal lobe left	-4.60 (-9.08, -0.12) 0.050	-2.76 (-7.48, 1.96) 0.258	-1.80 (-7.12, 3.52) 0.510
Parietal lobe right	-2.37 (-6.10, 1.36) 0.219	-3.89 (-7.99, 0.21) 0.069	-3.21 (-8.15, 1.74) 0.211
Occipital lobe left	-2.82 (-7.42, 1.77) 0.234	-4.61 (-9.10, -0.12) 0.050	-4.01 (-9.00, 0.98) 0.123
Occipital lobe right	-3.58 (-7.28, 0.12) 0.064	-2.36 (-6.10, 1.39) 0.223	-1.81 (-6.20, 2.59) 0.425
		**CN group**	
Cerebellum	1.32 (-0.28, 2.92) 0.110	0.91 (-0.80, 2.61) 0.3023	0.88 (-0.96, 2.71) 0.3544
Hippocampus	9.42 (-17.22, 36.06) 0.491	2.62 (-24.91, 30.16) 0.8528	5.43 (-23.54, 34.41) 0.7148
Hippocampus left	19.45 (-30.33, 69.22) 0.447	5.09 (-47.07, 57.24) 0.8492	10.21 (-44.61, 65.02) 0.7168
Hippocampus right	14.09 (-38.49, 66.67) 0.601	3.55 (-49.79, 56.90) 0.8967	8.55 (-47.43, 64.52) 0.7660
MTA	-0.00 (-0.12, 0.12) 0.958	0.02 (-0.11, 0.15) 0.7755	0.01 (-0.12, 0.15) 0.8436
MTA left	0.02 (-0.10, 0.14) 0.770	0.06 (-0.07, 0.20) 0.3695	0.06 (-0.08, 0.20) 0.4086
MTA right	-0.02 (-0.12, 0.09) 0.745	-0.01 (-0.12, 0.10) 0.8450	-0.02 (-0.13, 0.10) 0.7718
Temporal lobe left	-0.01 (-4.37, 4.35) 0.995	-0.57 (-5.10, 3.96) 0.8052	-0.60 (-5.30, 4.10) 0.8025
Temporal lobe right	0.45 (-4.17, 5.07) 0.849	-0.04 (-4.74, 4.66) 0.9860	-0.60 (-5.57, 4.36) 0.8132
Frontal lobe left	-0.72 (-3.44, 2.01) 0.609	-1.11 (-3.94, 1.72) 0.4461	-1.37 (-4.36, 1.62) 0.3731
Frontal lobe right	-0.34 (-3.05, 2.36) 0.805	-0.93 (-3.79, 1.93) 0.5273	-1.15 (-4.13, 1.83) 0.4529
Parietal lobe left	0.06 (-4.35, 4.47) 0.978	-1.06 (-5.66, 3.53) 0.6523	-0.56 (-5.49, 4.37) 0.8246
Parietal lobe right	-0.19 (-4.04, 3.66) 0.923	-1.97 (-6.30, 2.35) 0.3760	-1.69 (-6.23, 2.85) 0.4690
Occipital lobe left	-2.39 (-7.08, 2.30) 0.322	-2.15 (-6.83, 2.52) 0.3709	-1.70 (-6.75, 3.34) 0.5114
Occipital lobe right	-2.34 (-6.60, 1.92) 0.286	-1.83 (-6.12, 2.47) 0.4085	-1.68 (-6.24, 2.89) 0.4753

Model I adjusts for age, and sex; Model II adjusts for age, sex, BMI, hypertensive history, and diabetes history.

Bold fonts indicate they had statistical significance.

Supplementary Table 7. Association between Brain Volumes and Right Heel Strike Angle

	Crude	Model I	Model II
	β (95%CI) P		
**CI group**			
Cerebellum	0.87 (-1.07, 2.82) 0.383	1.44 (-0.54, 3.43) 0.161	1.11 (-1.09, 3.30) 0.328
Hippocampus	-14.97 (-37.31, 7.38) 0.195	-13.70 (-36.02, 8.62) 0.235	-10.11 (-34.45, 14.22) 0.420
Hippocampus left	-31.98 (-74.57, 10.61) 0.147	-28.75 (-71.42, 13.93) 0.193	-23.35 (-68.83, 22.14) 0.320
Hippocampus right	-24.10 (-67.90, 19.71) 0.286	-22.78 (-66.40, 20.84) 0.311	-14.30 (-62.61, 34.00) 0.565
**MTA**	**0.16 (0.06, 0.25) 0.003**	**0.15 (0.04, 0.25) 0.007**	**0.14 (-0.00, 0.28) 0.059**
**MTA left**	**0.13 (0.04, 0.23) 0.008**	**0.12 (0.02, 0.22) 0.024**	**0.10 (-0.03, 0.22) 0.142**
**MTA right**	**0.14 (0.05, 0.24) 0.004**	**0.14 (0.05, 0.24) 0.006**	**0.14 (0.01, 0.27) 0.045**
Temporal lobe left	-2.48 (-6.27, 1.30) 0.204	-2.14 (-5.94, 1.66) 0.275	-1.42 (-5.72, 2.87) 0.519
Temporal lobe right	-3.79 (-6.94, -0.64) 0.022	-3.34 (-6.63, -0.06) 0.052	-2.45 (-6.22, 1.33) 0.211
Frontal lobe left	-2.55 (-5.70, 0.60) 0.119	-2.14 (-5.53, 1.25) 0.222	-1.67 (-5.41, 2.07) 0.387
**Frontal lobe right**	**-5.37 (-8.43, -2.31) 0.001**	**-5.35 (-8.79, -1.92) 0.003**	**-5.39 (-9.46, -1.32) 0.013**
Parietal lobe left	-3.32 (-7.54, 0.91) 0.130	-2.87 (-7.19, 1.45) 0.199	-1.65 (-6.43, 3.14) 0.503
Parietal lobe right	-4.05 (-7.42, -0.69) 0.022	-3.71 (-7.47, 0.04) 0.059	-2.95 (-7.40, 1.49) 0.200
**Occipital lobe left**	**-5.41 (-9.46, -1.36) 0.011**	**-5.43 (-9.43, -1.43) 0.010**	**-5.19 (-9.52, -0.85) 0.024**
Occipital lobe right	-2.04 (-5.51, 1.43) 0.255	-2.10 (-5.54, 1.35) 0.238	-1.65 (-5.61, 2.30) 0.417
**CN group**			
Cerebellum	1.27 (-0.19, 2.73) 0.094	1.01 (-0.56, 2.57) 0.212	1.18 (-0.49, 2.84) 0.172
Hippocampus	11.11 (-13.23, 35.45) 0.375	6.44 (-18.91, 31.78) 0.620	10.55 (-15.88, 36.99) 0.437
Hippocampus left	25.23 (-20.13, 70.59) 0.280	16.08 (-31.83, 63.99) 0.513	23.48 (-26.41, 73.37) 0.360
Hippocampus right	14.44 (-33.71, 62.60) 0.559	6.57 (-42.61, 55.74) 0.794	13.98 (-37.24, 65.20) 0.595
MTA	0.01 (-0.10, 0.12) 0.918	0.01 (-0.11, 0.12) 0.906	-0.00 (-0.13, 0.12) 0.948
MTA left	0.03 (-0.08, 0.14) 0.614	0.04 (-0.08, 0.17) 0.498	0.03 (-0.10, 0.17) 0.611
MTA right	-0.01 (-0.11, 0.08) 0.810	-0.02 (-0.11, 0.08) 0.754	-0.03 (-0.13, 0.08) 0.625
Temporal lobe left	0.66 (-3.34, 4.65) 0.749	0.80 (-3.37, 4.98) 0.708	0.76 (-3.55, 5.07) 0.731
Temporal lobe right	1.77 (-2.44, 5.99) 0.413	1.84 (-2.46, 6.14) 0.405	1.38 (-3.16, 5.92) 0.554
Frontal lobe left	0.51 (-2.00, 3.01) 0.693	0.66 (-1.96, 3.28) 0.624	0.64 (-2.12, 3.40) 0.650
Frontal lobe right	0.19 (-2.30, 2.67) 0.883	0.16 (-2.48, 2.81) 0.904	0.16 (-2.59, 2.90) 0.912
Parietal lobe left	1.03 (-3.00, 5.07) 0.618	0.67 (-3.58, 4.91) 0.759	1.53 (-2.97, 6.03) 0.508
Parietal lobe right	0.67 (-2.86, 4.19) 0.712	0.16 (-3.86, 4.18) 0.936	0.52 (-3.66, 4.70) 0.807
Occipital lobe left	-0.98 (-5.31, 3.36) 0.660	-0.63 (-4.97, 3.72) 0.778	-0.21 (-4.86, 4.43) 0.929
Occipital lobe right	-3.05 (-6.91, 0.81) 0.127	-2.56 (-6.49, 1.37) 0.207	-2.63 (-6.77, 1.50) 0.218

Model I adjusts for age, and sex; Model II adjusts for age, sex, BMI, hypertensive history, and diabetes history.

Bold fonts indicate they had statistical significance.
